# Editorial: Emergency medicine in pediatric patients with chronic diseases

**DOI:** 10.3389/fped.2023.1185636

**Published:** 2023-03-30

**Authors:** Emmanuel Schneck, Johannes Wolff, Jan-Thorsten Graesner, Holger Hauch

**Affiliations:** ^1^ Department of Anesthesiology, Intensive Care Medicine and Pain Therapy, Justus Liebig University Giessen, Giessen, Germany; ^2^ Department of Oncology, Cleveland Clinic, Pediatric Oncology, Cleveland, OH, United States; ^3^ Department of Anesthesiology and Intensive Care Medicine, Institute of Emergency Medicine, University Hospital Schleswig-Holstein, Kiel, Germany; ^4^ Department of Pediatric Oncology and Hematology, Justus Liebig University Giessen, Giessen, Germany

**Keywords:** palliative, emergency medical service (EMS), rare disease, children, emergency care

**Editorial on the Research Topic** Emergency medicine in pediatric patients with chronic diseases

The editors are pleased to introduce this special issue of Frontiers in Pediatrics, which includes eight articles discussing emergency medicine in pediatric patients with chronic diseases. The life expectancy of children with chronic diseases is improving; due to an increasing number of innovative therapies, pediatric patients can now survive formerly life-threatening diseases (1, 2). In most cases, however, these children remain chronically ill and continue to have debilitating symptoms and specific, complex needs. Care for these patients demands highly educated teams and specialists (3). However, in emergencies, they must be treated by emergency medical services (EMS) and non-specialized physicians leaving both patients and healthcare providers in a challenging situation.

For this reason, this special issue aims to shed some light on different scenarios of chronically ill children in emergency settings. In total, this research topic includes three original articles, one brief scientific research letter, two case reports, and two reviews.

## Pre-hospital management of critical illness in pediatric patients with chronic diseases

To begin this section, Uzun et al. give an overview of cardiopulmonary resuscitation in chronically ill children. Since general guidelines do not offer specific instructions for this special patient population, valuable information and recommendations are provided in this review. In summary, 20 eligible publications focusing on children with cardiac and cancerous diseases who suffered from cardiac arrest were included to the review. The authors concisely summarize the complex factors which must be considered during the resuscitation of chronically ill children. Schneck et al. present a multicentric analysis of 880 pediatric palliative patients of which 17 were resuscitated during cardiac arrest despite receiving treatment from specialist home palliative care teams. Their investigation focuses on the decision-making process leading to resuscitation attempts in these children and shows a surprisingly high survival rate. One outstanding question from this study is how to prepare parents and EMS teams for these challenging situations. Hauch et al. address this within their cross-sectional, exploratory study by interviewing more than 1,000 EMS providers about their needs and worries when encountering children receiving palliative home care. The resulting findings strongly suggest a need for specialized training in this field.

## In-hospital management of critical illness in pediatric patients with chronic and rare diseases

In the following section, the focus switches from pre- to in-hospital settings. Agarwal et al. give in their review article an insightful overview of medical emergencies during and after pediatric blood and bone marrow transplantation and cellular therapies. Pediatric patients are particularly at risk for severe complications that require urgent management after bone marrow transplants. Despite this review displays not a systemic review or meta-analysis, the authors were able to present a concise review on typical complications of bone marrow transplantation with a strong focus on their management.

The following articles address emergencies in children with rare diseases. In a brief research letter, Sánchez-Pintos et al. discuss their retrospective study on the use of intravenous branched-chain amino acid-free solutions to treat metabolic decompensation episodes in five patients with maple syrup urine disease. The results show that these intravenous solutions might offer an additional alternative in critically ill children. A retrospective, observational, singe-center analysis by Icheva et al. focuses on 15 infants with systemic-to-pulmonary shunts and acquired von Willebrand syndrome. Although von Willebrand syndrome is known to be connected to congenital heart disease, this study underlines its high incidence in patients with systemic-to-pulmonary shunts as well as its need for a specific diagnostic test and coagulatory therapy.

Finally, two case reports give interesting insights into emergencies in pediatric patients with rare conditions. In their article, Mand et al. report two cases of rhabdomyolysis in children, one in a formerly healthy and one in a chronically ill child. The case report highlights the warning signs of rhabdomyolysis alongside the necessary therapeutic approaches. Schumann et al. present a further case series on four successful cases of post-pyloric nutrition for the prevention of metabolic decompensation in children with methylmalonic and propionic acidemia. Even though these diseases are very rare, the development of catabolism and acidosis indicates a life-threatening condition in these patients and can occur even during minor infections. For this reason, the knowledge of prevention strategies including post-pyloric nutrition is of high importance.

This special issue summarizes knowledge on pre- and in-hospital emergency medicine in children with chronic diseases and the editors hope that it will be an interesting, educational read.
